# Ertapenem-Associated Neurotoxicity in a Patient With Chronic Kidney Disease and Hypothyroidism: A Case Report

**DOI:** 10.7759/cureus.112771

**Published:** 2026-07-16

**Authors:** Manar Elfatih Mohamed, Esraa Abdulla Ibrahim Ockba, Arshee Khan

**Affiliations:** 1 Internal Medicine, Dubai Health, Dubai, ARE; 2 Internal Medicine, Dubai Academic Health Corporation, Dubai, ARE

**Keywords:** chronic kidney disease (ckd), ertapenem, hypoalbuminemia, hypothyroidism, seizures

## Abstract

Carbapenems are broad-spectrum antibiotics used to treat infections caused by multidrug-resistant organisms and are associated with neurotoxicity, especially in high-risk groups. Although ertapenem generally has a lower risk of seizures, factors such as advanced age, pre-existing neurological conditions, renal impairment, and hypoalbuminemia can raise the likelihood of adverse effects. We report a 92-year-old female with multiple comorbidities, including hypothyroidism, dementia, well-controlled post-stroke epilepsy, and chronic kidney disease, who developed a focal impaired awareness seizure on day 3 of IV ertapenem treatment for a multidrug-resistant urinary tract infection, despite prior tolerance to intravenous ertapenem, with complete resolution after discontinuation of IV ertapenem. This case highlights that ertapenem can be a potential precipitating factor for neurotoxicity in patients with a history of stable epilepsy and tolerance to the drug, particularly in those with advanced age and chronic kidney disease. Early recognition and discontinuation of the antibiotic are essential, as neurological symptoms are typically reversible.

## Introduction

Carbapenems constitute a class of broad-spectrum antibiotics. β-lactam antibiotics are primarily used to treat infections caused by multidrug-resistant pathogens, particularly in hospital settings, and are often reserved as last-line therapies. Carbapenems are resistant to β-lactamase degradation, thus offering extensive antimicrobial activity against extended-spectrum β-lactamase (ESBL)-producing organisms and various Gram-negative bacteria [1]. Although highly efficacious, carbapenems are associated with proconvulsant and epileptogenic effects. Imipenem presents the highest risk of seizures (3-33%), whereas doripenem and ertapenem carry a lower risk (<1%), with approximately 0.18% of cases reported to be related to ertapenem-induced seizures [1]. Ertapenem is predominantly eliminated via renal excretion, consequently elevating the risk of neurotoxicity in patients with impaired renal function and those with chronic kidney disease (CKD) [2]. Ertapenem-associated neurotoxicity is thought to result from gamma-aminobutyric acid A (GABA-A) receptor inhibition, leading to increased neuronal excitability, with additional patient-related factors, including advanced age, pre-existing neurological disease, hypoalbuminemia, and thyroid dysfunction, potentially contributing to susceptibility [3]. While the neurotoxicity induced by ertapenem is well documented, most reports describe a single episode of exposure with subsequent symptom resolution upon discontinuation of the drug [3,4]. Data regarding the effects of repeated ertapenem exposure and potential alterations in susceptibility over time remain limited.

## Case presentation

A 92-year-old female with a history of ischemic heart disease, heart failure with preserved ejection fraction, atrial fibrillation, chronic kidney disease, hypothyroidism, dementia, and well-controlled post-stroke epilepsy on phenytoin presented with symptoms suggestive of a urinary tract infection. Urine cultures grew multidrug-resistant ESBL-producing *Escherichia coli* and *Klebsiella* species, and she was started on a renal-adjusted dose of intravenous ertapenem 500 mg once daily.

**Figure 1:**
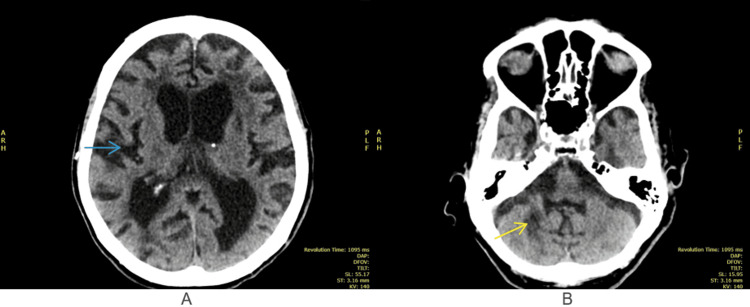
CT of the brain demonstrating (A) advanced degenerative atrophy (blue arrow), and (B) old right cerebellar cerebrovascular accident (yellow arrow).

On day 3 of therapy, she developed facial twitching, right upper limb myoclonic jerks, and a brief episode of unresponsiveness lasting approximately one minute, followed by post-ictal confusion. She had been seizure-free for the preceding year on phenytoin 100 mg twice daily. Her epilepsy was initially diagnosed 10 years earlier after status epilepticus following a cerebrovascular accident, and she remained seizure-free for approximately nine years. A previous urinary tract infection treated with oral ciprofloxacin triggered a focal impaired awareness seizure, leading to the reintroduction of antiepileptic medication after six years off medication. Brain computed tomography (CT) demonstrated an old right cerebellar cerebrovascular accident with focal gliotic changes and advanced degenerative atrophy, without evidence of recent infarction, hemorrhage, or mass lesion (Figure 1). Three months later, she developed another multidrug-resistant urinary tract infection and was treated with IV ertapenem. On day 6 of therapy, she experienced two self-limited focal impaired awareness seizures, which resolved completely after discontinuation of ertapenem while her regular phenytoin dose was continued. Notably, she had received ertapenem three years earlier for a urinary tract infection without adverse neurological effects.

**Figure 2:**
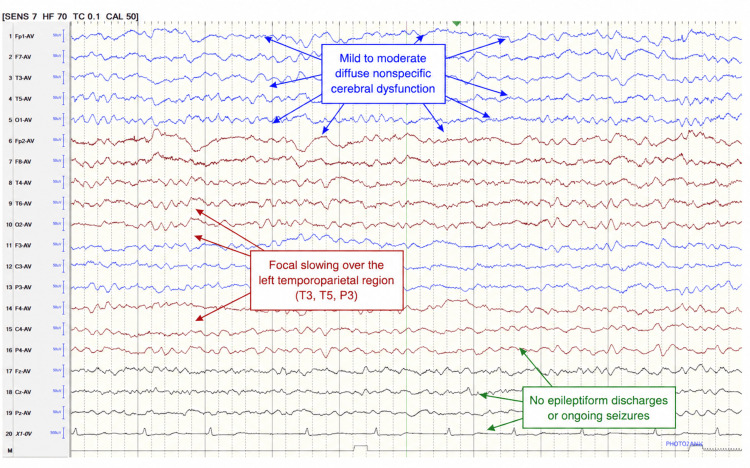
EEG showing mild to moderate nonspecific cerebral dysfunction, diffuse and focal over the left temporoparietal region, without epileptiform discharges or ongoing seizures.

Laboratory investigations revealed normal electrolyte levels and borderline low albumin (Table 1). However, her serum phenytoin level was subtherapeutic (1.7 μg/mL). Electroencephalography (EEG) showed mild to moderate nonspecific cerebral dysfunction, diffuse and focal over the left temporoparietal region, without epileptiform discharges or ongoing seizures (Figure 2).

**Table 1:** Laboratory investigations.

Parameters	Patient values	Reference range
Hemoglobin	10.6	12-15 g/dL
Platelets	140	150-410 x10^3 /µL
WBC count	7.1	3.6-11.0 x10^3 /µL
Creatinine	1.69	0.70-1.20 mg/dL
Estimated glomerular filtration rate	28.3	>60 ml/min/1.73 m^2^
Sodium	137	136-145 mmol/L
Potassium	4.4	3.4-4.5 mmol/L
Urea	165	12-40 mg/dL
Bicarbonate	18.9	20-28 mmol/L
Bilirubin	0.37	0-1.0 mg/dL
Alanine transaminase	33	0-31 U/L
Aspartate transaminase	30	0-32 U/L
Alkaline phosphatase	119	35-104 U/L
Albumin	3.7	3.9-5.0 g/dL
Thyroid-stimulating hormone (TSH)	4.650	0.27-4.20 uIU/mL
Free T4	8.0	12-22 pmol/L
Calcium	9.0	9.0-10.2 mg/dL
Phosphate	3.4	2.7-4.5 mg/dL
Magnesium	2.22	1.7-2.3 mg/dL
C-reactive protein	42.3	<5.0 mg/L
Procalcitonin	0.24	<0.05 ng/mL
Phenytoin	1.70	10-20 ug/mL

Ertapenem was discontinued, and her phenytoin dose was increased to 200 mg twice daily, after which she remained seizure-free. At three-week follow-up, her phenytoin level was supratherapeutic, and the dose was reduced to 100 mg twice daily. A temporal pattern of progressively earlier seizure onset with repeated ertapenem exposure was noted in our patient, as summarized in Table 2.

**Table 2:** Temporal trends in phenytoin levels, renal function, and thyroid function in relation to episodes of ertapenem-associated seizures. TSH: thyroid-stimulating hormone; eGFR: estimated glomerular filtration rate; BID: twice per day.

Date	Neurological manifestation	Ertapenem exposure	Phenytoin level (ug/mL)	Antiepileptic	TSH (uIU/mL)	T4 (pmol/L)	Albumin (g/dL)	eGFR (mL/min/1.73 m^2^)
November 2021	Neurologically stable	Completed 5 days	-	None	4.00	17.8	4.0	23.3
January 2025	Facial twitching and up-rolling of the eyes	Day 6	2.20	Phenytoin 100 mg BID	4.48	8.5	3.2	34.9
Early November 2025	Facial twitching, jerky movements of the right arm, and a brief period of unresponsiveness lasting approximately one minute	Day 3	1.7	Phenytoin 100 mg BID	4.65	8	3.6	34.6
Follow-up								
Late November 2025	Neurologically stable	-	24	200 mg BID				
December 2025	Neurologically stable	-	2.95	100 mg BID	5.38	8.2	3.7	30

## Discussion

Ertapenem-associated neurotoxicity is most commonly observed in patients receiving high-dose therapy, older patients, those with pre-existing central nervous system (CNS) pathology, or those with impaired renal function, as observed in our patient [2,5]. Renal impairment leads to accumulation and increased CNS penetration, resulting in inhibition of GABA-A receptor activity even with dose adjustments [6,7]. Although our patient received a renal-adjusted dose of intravenous ertapenem and her renal function remained stable throughout the treatment courses, she developed focal impaired awareness seizures, highlighting that neurotoxicity may still occur despite appropriate dosing. Symptoms typically develop within two to 10 days of therapy and often resolve after discontinuation of the drug, regardless of dialysis [5]. Prolonged ertapenem-induced neurotoxicity, such as encephalopathy, has been described in the literature in patients with renal impairment [8].

An important observation in our patient was that the time from the initiation of ertapenem therapy to the onset of seizure progressively shortened with repeated ertapenem exposure, decreasing to three days by the most recent course, as shown in Table 2. This pattern may explain an increased susceptibility to ertapenem-induced neurotoxicity over time, even in patients with previously stable seizure control. Although the underlying mechanism remains unclear, this observation raises the possibility of cumulative susceptibility, a phenomenon not well described in the literature and requiring further investigation. Recent studies have characterized the clinical spectrum of ertapenem neurotoxicity. A review of 66 patients identified seizures as the most common presentation (42.4%), followed by visual hallucinations, altered mental status, confusion, and disorientation, with rarer symptoms including cognitive changes, dysarthria, and sleepwalking [3]. A 2024 systematic review of 125 cases reported a median onset of four days, with seizures occurring in 70% of patients [4]. A meta-analysis looked at the effects of antiepileptic drugs on thyroid function and found that phenytoin is associated with significant reductions in total and free thyroxine (T4) levels, primarily due to hepatic enzyme induction and altered thyroid hormone metabolism. However, overt clinical hypothyroidism is uncommon [9]. Furthermore, the mild hypothyroidism seen in our patient might have contributed to reduced renal clearance and increased risk of drug accumulation [10]. In addition, ertapenem has been shown to decrease anticonvulsant drug levels, including valproate, potentially reducing seizure control and precipitating breakthrough seizures [11]. Hypoalbuminemia has been shown to elevate ertapenem neurotoxicity by elevating the free drug concentrations, enhancing cerebrospinal fluid penetration, and potentially triggering neuropsychiatric complications [3,12]. The degree of hypoalbuminemia in our patient was mild. Similarly, mild hypothyroidism was considered only a possible contributing factor rather than an independent cause of neurotoxicity. Chronic kidney disease likely represented the most significant risk factor in our patient. A 2021 retrospective study reported that the current guidelines' approved dose of ertapenem may increase the risk of neurotoxicity in patients with end-stage renal disease [2]. Further large-scale studies in the field of antibiotic toxicity are needed to accurately study the incidence and clinical outcomes of ertapenem-associated neurotoxicity.

Although phenytoin dose escalation may have contributed to seizure control, the concurrent discontinuation of ertapenem and subsequent clinical improvement suggest that removal of the potential offending agent played an important role in recovery. Multiple patient-related factors may have contributed to lowering the seizure threshold in our patient, including advanced age, chronic kidney disease, structural brain disease, infection, and subtherapeutic phenytoin levels. However, the temporal pattern of onset, recurrence with re-exposure, and recovery strongly supports ertapenem as the most likely trigger.

## Conclusions

Our case suggests that ertapenem was the most likely precipitating factor for breakthrough seizures in a patient with long-standing, well-controlled epilepsy, highlighting that ertapenem-associated neurotoxicity may occur despite prior tolerance to the drug. Uniquely, our patient exhibited progressively shorter time to seizure onset with repeated ertapenem courses, suggesting increased susceptibility over time, a phenomenon not previously reported in the literature to the best of our knowledge. There are several contributing factors, likely including chronic kidney disease, structural CNS lesions, mild hypothyroidism, and subtherapeutic phenytoin levels. Patients at risk will require close and regular monitoring of renal function as well as serum albumin, timely dose adjustments, and early detection of neurological symptoms to prevent ertapenem-induced seizures. Early recognition and prompt discontinuation often result in complete resolution of seizures, emphasizing the importance of vigilance and individualized management in high-risk patients.
